# Variation in the Ovine KAP6-3 Gene (*KRTAP6-3*) Is Associated with Variation in Mean Fibre Diameter-Associated Wool Traits

**DOI:** 10.3390/genes8080204

**Published:** 2017-08-18

**Authors:** Shaobin Li, Huitong Zhou, Hua Gong, Fangfang Zhao, Jiqing Wang, Yuzhu Luo, Jon G. H. Hickford

**Affiliations:** 1 Gansu Key Laboratory of Herbivorous Animal Biotechnology, Faculty of Animal Science and Technology, Gansu Agricultural University, Lanzhou 730070, China; lisb@gsau.edu.cn (S.L.); zhouh@lincoln.ac.nz (H.Z.); zhaoff@gsau.edu.cn (F.Z.); wangj@gsau.edu.cn (J.W.); 2 International Wool Research Institute, Gansu Agricultural University, Lanzhou 730070, China; hua.gong@lincoln.ac.nz; 3 Gene-marker Laboratory, Faculty of Agricultural and Life Sciences, Lincoln University, Lincoln 7647, New Zealand

**Keywords:** KAP6-3 gene (*KRTAP6-3*), mean fibre diameter (MFD), wool traits, sheep

## Abstract

Polymerase chain reaction-single stranded conformational polymorphism (PCR-SSCP) analysis was used to investigate variation in the ovine KAP6-3 gene (*KRTAP6-3*) in 383 Merino × Southdown-cross lambs from four sire-lines, and to determine whether this variation affects wool traits. Five PCR-SSCP banding patterns, representing five different nucleotide sequences, were detected, including four previously identified (named *A*, *B*, *C,* and *F*) variants and one newly identified (named *G*) variant. A new non-synonymous single nucleotide polymorphism (SNP) and a 45-bp deletion were detected in variant *G*. Of the three common genotypes (*AA*, *AB,* and *AG*) identified in these sheep, wool from sheep that were *AG*, on average, had a lower mean fibre diameter (MFD), fibre diameter standard deviation (FDSD), and prickle factor (PF) than wool from *AA* sheep, whereas wool from *AB* sheep, on average, had a higher MFD, FDSD, and PF than wool from *AA* sheep. This suggests that variation in ovine *KRTAP6-3* affect MFD, FDSD, and PF, and that this gene may have potential for use as a gene-maker for improving fibre diameter-associated wool traits.

## 1. Introduction

Sheep have been bred for wool production for thousands of years and globally a large diversity of wool sheep breeds have been created to meet different market demands. To increase the speed at which sheep with desirable wool characteristics can be bred, while at the same time reducing fleece to fleece and fibre to fibre variation, emphasis has begun to focus on the proteins, and thus genes, that are involved in producing the wool fibre.

Keratin-associated proteins (KAPs) are structural components of the wool fibre. They create a semi-rigid matrix around the keratin intermediate filaments (KIFs) and play an important role in defining the physico-mechanical properties of the fibres [1]. KAPs are a complex class of proteins and they typically possess a high level of cysteine, or both glycine and tyrosine. They have been allocated into three broad groups according to their amino acid composition: the high sulphur (HS: ≤30 mol% cysteine) KAPs, the ultra-high sulphur (UHS: >30 mol% cysteine) KAPs, and the high glycine-tyrosine (HGT: 35–60 mol% glycine and tyrosine) KAPs [2]. The KAP proteins are encoded by a large number of genes designated as *KRTAPs*. More than 100 *KRTAPs* have been identified across species and these have been divided into 27 KAP families [3,4,5]. Of these, families KAP1 to 3, 11, 13 to 16, and 23 to 27 are HS-KAPs; families 4, 5, 9, 10, 12, and 17 are UHS-KAPs, and families 6 to 8 and 18 to 22 are HGT-KAPs.

The HGT-KAPs are predominantly found in the orthocortex of wool fibre and they are the first KAPs expressed after the synthesis of the KIFs. Different wools vary considerably in their HGT-KAP content, ranging from less than 1% by weight in the wool of Lincoln sheep, to between 4% and 12% by weight in the wool of Merinos [6]. A reduction in the content of HGT-KAPs appears, at least in part, to be responsible for the felting lustre mutant found in Merino sheep [7]. The wide range in the proportional content of HGT-KAPs in wool from different breeds raises intriguing questions about the function of these proteins in fibre characteristics.

To date, four families of HGT-KAPs have been identified in sheep, and these are the KAP6, KAP7, KAP8, and KAP22 families [2,8]. KAP7 and KAP22 contain a single family member, and only two family members have been described for KAP8 [2,8]. In contrast, KAP6 appears to be a diverse family, with five family members having been described in sheep, and all of the genes being polymorphic [9]. Furthermore, two of the KAP6 genes (*KRTAP6-1* and *KRTAP6-3*) have been revealed to have length variation [9]. With *KRTAP6-1* this variation has been found to be associated with fibre-diameter-associated traits [10]. The effect of *KRTAP6-3* variation on wool traits is still unknown.

The objective of this study was to investigate *KRTAP6-3* variation in a Merino × Southdown-cross flock, and to ascertain whether any genetic variation found was associated with variation in wool traits including greasy fleece weight (GFW), clean fleece weight (CFW), wool yield (Yield), mean fibre diameter (MFD), fibre diameter standard deviation (FDSD), coefficient of variation of fibre diameter (CVFD), mean staple length (MSL), mean fibre curvature (MFC), mean staple strength (MSS), and prickle factor (PF).

## 2. Materials and Methods

### 2.1. Sheep Blood and Wool Samples

Three hundred and eighty-three Merino × Southdown-cross lambs farmed at Ashley Dene (Lincoln University, Canterbury, New Zealand), and from four sire-lines, were investigated. All lambs were ear-tagged with a unique identification number within 12 h of birth and their birth dates, birth weights, birth ranks (i.e., whether they were a single, twin, or triplet), genders, and dam numbers were recorded. All of the ewes and lambs were brought together at tailing (lambs aged between two and six weeks old) and remained together until weaning. At tailing, blood samples from all these sheep were collected onto FTA cards (Whatman BioScience, Middlesex, UK) and genomic DNA was purified using a two-step procedure described by Zhou et al. [11].

Wool samples were collected at 12 months of age (first shearing) from the mid-side of the lambs. GFW was measured at shearing and Yield, MFD, FDSD, CVFD, MSL, MFC, MSS, and PF were measured by the New Zealand Wool Testing Authority (NZWTA) (Ahuriri, Napier, New Zealand). Wool traits were measured using International Wool Textile Organisation (IWTO) measurement standards (NZWTA, IWTO License N° 3. Test-Methods for which Licensed: IWTO-6, -7, -10, -12, -17, -19, -28, -30, -31, -33, -38, -47, and -56). CFW was calculated from the GFW and Yield measurements (i.e., Yield = CFW/GFW × 100%).

### 2.2. PCR Primers and Amplification

Two polymerase chain reaction (PCR) primers, 5′-CCGAGAACAACCTCAACTAC-3′ and 5′-GTAGAGGATGAGAGTCTTTCT-3′, were designed to amplify a variable region (c.-27 to c.*29) of *KRTAP6-3*, based on the published *KRTAP6-3* sequences and a comparison with other *KRTAP6-n* sequences [9]. The primers were synthesised by Integrated DNA Technologies (Coralville, IA, USA). PCR amplification was performed in a 15-μL reaction containing the genomic DNA on one 1.2-mm punch of FTA paper, 0.25 μM of each primer, 150 μM of each deoxynucleotide (dNTP) (Bioline, London, UK), 2.5 mM of Mg^2+^, 0.5 U of Taq DNA polymerase (Qiagen, Hilden, Germany), and 1× reaction buffer supplied with the enzyme. The thermal profile consisted of 2 min at 94 °C, followed by 35 cycles of 30 s at 94 °C, 30 s at 60 °C, and 30 s at 72 °C, with a final extension of 5 min at 72 °C. Amplification was carried out using S1000 thermal cyclers (Bio-Rad, Hercules, CA, USA).

Amplicons were visualized by electrophoresis in 1% agarose gels (Quantum Scientific, Brisbane, Queensland, Australia), using 1× Tris/Borate/EDTA (TBE) buffer [89 mM Tris, 89 mM boric acid, and 2 mM ethylenediaminetetraacetic acid disodium salt (EDTA-Na_2_)] containing 200 ng/mL of ethidium bromide.

### 2.3. Screening for Variation in *KRTAP6-3*

The PCR amplicons were screened for sequence variation using single stranded conformational polymorphism (SSCP) analysis. A 0.7-μL aliquot of each amplicon was mixed with 7 μL of loading dye (98% formamide, 10 mM EDTA, 0.025% bromophenol blue, 0.025% xylene-cyanol). After denaturation at 95 °C for 5 min, the samples were rapidly cooled on wet ice and then loaded on 16 cm × 18 cm, 12% acrylamide: bisacrylamide (37.5:1) (Bio-Rad) gels containing 3.5% *v*/*v* glycerol. Electrophoresis was performed using Protean II xi cells (Bio-Rad) in 0.5× TBE buffer, under the electrophoretic conditions of 17 °C, 350 V for 18 h. Gels were silver-stained according to the method of Byun et al. [12].

### 2.4. Sequencing of Allelic Variants and Sequence Analysis

PCR amplicons representing different banding patterns from sheep that appeared to be homozygous were sequenced in both directions at the Lincoln University DNA sequencing facility, New Zealand. Alleles that were only found in heterozygous sheep were sequenced using an approach described by Gong et al. [13]. Briefly, a band corresponding to the allele was excised as a gel slice form the polyacrylamide gel, macerated, and then used as a template for re-amplification with the original primers. This second amplicon was then sequenced.

Sequence alignments and translations were carried out using DNAMAN (version 5.2.10, Lynnon BioSoft, Vaudreuil, QC, Canada). Potential phosphorylation sites were predicted using the NetPhos 3.1 Server (www.cbs.dtu.dk/services/NetPhos/).

### 2.5. Statistical Analyses

Statistical analyses were performed using Minitab version 16 (Minitab Inc., State College, PA, USA). General linear models (GLMs) were used to compare the various wool traits in sheep of different genotypes and with a Bonferroni correction being applied to reduce the chances of obtaining false positive results during the repeated comparisons. Sire was found to affect (*p* < 0.05) all the wool traits, and gender was also found to affect (*p* < 0.05) or potentially affect (*p* < 0.20) the wool traits; hence, both sire and gender were included as explanatory factors in the models. Birth rank was not found to affect or potentially affect wool traits, and was not factored into the models.

## 3. Results

### 3.1. Variation in Ovine *KRTAP6-3*

**Figure 1:**
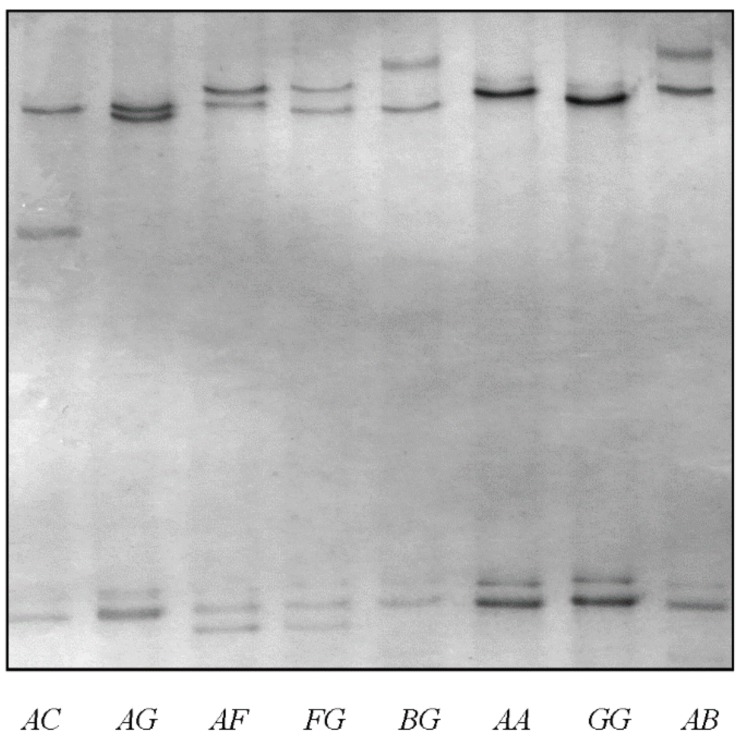
Polymerase chain reaction-single stranded conformational polymorphism (PCR-SSCP) analysis of ovine *KRTAP6-3.* Five banding patterns, representing five variants (*A*, *B*, *C*, *F,* and *G*) were identified in either homozygous or heterozygous forms in the sheep investigated.

In the 383 Merino × Southdown-cross lambs, five PCR-SSCP banding patterns were detected for *KRTAP6-3*, with either one or a combination of two banding patterns being observed for each sheep (Figure 1). DNA sequencing revealed that the banding patterns represented five different nucleotide sequences. Four of them were identical to previously reported *KRTAP6-3* sequences (GenBank KT725833, KT725834, KT725835, and GU319876, or variants *A*, *B*, *C,* and *F* respectively) [9,14], whereas the remaining sequence was unique, but shared a high degree of sequence similarity with the known *KRTAP6-3* sequences. This suggested that the unique sequence was a new variant form of ovine *KRTAP6-3*. Two other previously reported *KRTAP6-3* sequences (*D* and *E*) were not detected in these sheep [9], but the newly identified variant was named *G*, and its sequence submitted to GenBank (accession number MF061690).

**Figure 2:**
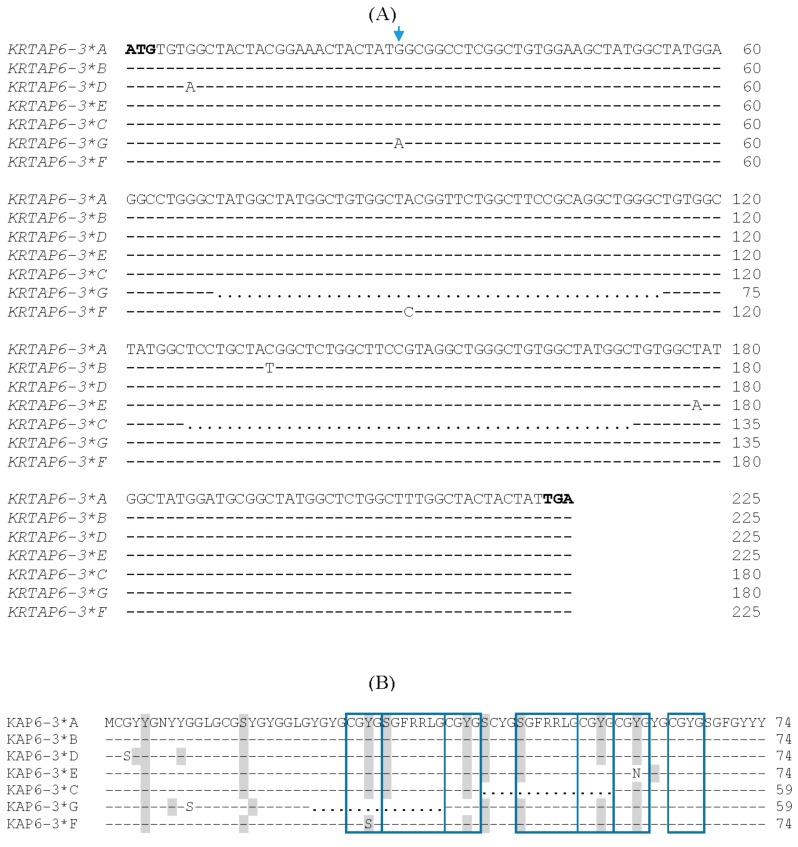
Sequence comparisons of the newly identified and previously described variants of ovine *KRTAP6-3*. (**A**) Alignment of the nucleotide sequences revealed five single nucleotide polymorphisms (SNPs) and two 45-bp deletions in the gene; (**B**) These nucleotide sequence variations would lead to four amino acid changes and two deletions of 15 amino acids in the central region of the putative protein, which contains repeats of the sequences CGYG and SGFRRLG. Nucleotides or amino acid residues identical to the top sequence are indicated by dashes, and dots represent deletions. The arrow indicates the new SNP identified in this study, and the boxes indicate repeats of amino acid motifs. Residues that could be phosphorylated are shaded.

A sequence comparison of the newly identified variant (*G*) and the six previously identified (*A* to *F*) variants revealed a new single nucleotide polymorphism (SNP) (c.28G/A) and a 45-bp deletion (c.70_114del) in ovine *KRTAP6-3* (Figure 2A). The SNP was non-synonymous and would result in an amino acid substitution (p.Gly10Ser) (Figure 2B). The deletion would notionally result in the loss of 15 amino acids in the central region of the protein. This region contains sequence repeats of CGYG and SGFRRLG (Figure 2B).

Among the sheep investigated, eight genotypes were detected and these were *AA*, *AB*, *AC*, *AG*, *AF*, *GG*, *BG,* and *FG*. The most common genotype was *AA* (61.7%), and the next most common genotypes were *AG* (27.4%) and *AB* (4.4%). The other five genotypes (*AC*, *AF*, *BG*, *FG,* and *GG*) were rare. The frequency of the individual variants was 79.8%, 2.6%, 0.3%, 1.7%, and 15.6%; for *A*, *B*, *C*, *F*, and *G*, respectively.

### 3.2. Associations between Variation in *KRTAP6-3* and Wool Traits

As genotypes *AC*, *AF*, *BG*, *FG,* and *GG* were rare in the sheep investigated, these genotypes were not investigated in the association analyses. With the three common *KRTAP6-3* genotypes (*AA*, *AB,* and *AG*), an effect of genotype was observed for MFD, FDSD, and PF (Table 1). Wool from sheep of genotype *AG* had lower average MFD, FDSD, and PF than wool from sheep of genotype *AA* (*p* < 0.01), whereas wool from sheep of genotype *AB* had higher average MFD, FDSD, and PF than wool from sheep of genotype *AA* (*p* < 0.01). This suggests that variation in ovine *KRTAP6-3* affects MFD, FDSD, and PF, with *G* being associated with a decrease and *B* being associated with an increase in these traits.

## 4. Discussion

This study describes genetic variation in ovine *KRTAP6-3* and its association with some wool traits in Merino × Southdown-cross sheep. Five variants, including four previously identified and one newly identified variant of ovine *KRTAP6-3* were detected, and variation in this gene was found to be associated with variation in three fibre diameter-associated wool traits: MFD, FDSD, and PF. The detection of associations with these three wool traits is consistent with the observation that these traits are strongly correlated [15].

The effect of *KRTAP6-3* on wool traits detected in this study is consistent with the previous finding of a quantitative trait locus (QTL) on chromosome 1 for MFD in medium wool Merinos [16], and is similar to the finding reported for *KRTAP6-1* [10] in which variation in that gene is also found to be associated with variation in MFD, FDSD, and PF. This is probably not surprising given that both genes are clustered next to each other on sheep chromosome 1, and that their sequences are more similar to each other than to any other *KRTAP6-n* [9].

Despite the observation that both *KRTAP6-3* and *KRTAP6-1* are polymorphic, the nature of the SNPs detected in these genes appears to be different. With *KRTAP6-1*, the SNPs are located outside the coding region [10], but with *KRTAP6-3*, all of the SNPs are located in the coding regions and the majority of them (four out of five) are non-synonymous. The common feature shared by these two genes appears to be the occurrence of length variation in the central region of the coding sequence. A deletion of 57-bp is observed in variant *C* of *KRTAP6-1,* and this deletion is found to associated with fibre diameter-associated wool traits [10]. In this study, a deletion of 45-bp is detected in variants *C* and *G* of *KRTAP6-3*, and *G* is found to be associated with variation in the same wool traits. This suggests that length variation may have an effect on wool traits.

The length variation in *KRTAP6-3* may affect wool traits in many ways. Firstly, the deletion found in variants *C* and *G* may result in the loss of a 15 amino acid string that contains repeat sequences in the central region of the protein (Figure 2B). This may have an effect on the structure and/or folding of the protein. Secondly, the deletion will result in a 15–20% reduction in the occurrence of aromatic amino acid residues (tyrosine and phenylalanine) and a 50% reduction in the occurrence of basic amino acid residues (arginine) in the protein (Figure 2B). Given that aromatic and basic amino acids are involved in the formation of cation-π interactions [17], a reduction in the number of these residues may have an impact on the strength of interaction between KAP6-3 and the KIF proteins. Thirdly, the 45-bp deletion would also lead to the loss of cysteine and glycine residues (Figure 2). Cysteine is essential for the formation of disulphide bonds. It is also vital for wool growth and is usually the first-limiting amino acid for wool fibre synthesis. While the precise role of glycine in HGT-KAPs has not been established, glycine is the smallest amino acid and lacks a side chain. This may make the HGT-KAPs more flexible and thus better able to form a compact structure with KIF proteins. Lastly, the deletion may also lead to a reduction in the number of residues that might be phosphorylated. This may result in changes in the solubility of keratins, in the organization of keratin filaments, and in the interactions with other proteins [18,19], and consequently influence wool traits.

Despite the same length (45 bp) of deletion being described in variant *C* [9], the deletion identified in variant *G* appears to be novel and located at a different position (Figure 2). Deletions/insertions that maintain the reading frame have been reported for other ovine *KRTAPs*, such as *KRTAP1-1* [20], *KRTAP5-4* [21], and *KRTAP 6-1* [10]. The identification of this new deletion in *KRTAP6-3* supports the notion that length variation is a structural hallmark of the *KRTAPs* [10].

The effect of *KRTAP6-3* variation on wool traits may also be due to the non-synonymous SNPs. It is notable that three out of the four non-synonymous SNPs detected in ovine *KRTAP6-3* would lead to amino acid substitutions of either glycine or tyrosine, by serine (Figure 2B). This type of substitutions has not been observed in any other ovine HGT-*KRTAP*, including *KRTAP6-1* [10], *KRTAP7-1* [22], *KRTAP8-1* [22], *KRTAP8-2* [23], or *KRTAP 22-1* [8]. However, the presence of SNPs leading to the loss or gain of serine has been reported in other HS- and UHS-*KRTAPs*, including *KRTAP5-4* [21] and *KRTAP15-1* [24]. The significance of the substitution of either glycine or tyrosine with serine is unknown, but given that both glycine and tyrosine are the characteristic residues of the HGT-KAPs, and that analysis suggests that many of these substitutions may affect the phosphorylation status of residues nearby (Figure 2B), it is logical that these SNPs may have an effect on the structure of the KAP6-3 proteins and/or their assembly.

The results from this study also suggest synonymous SNPs may have a functional effect. Given that the only difference between variants *A* and *B* is a single synonymous SNP, the difference in wool traits between genotypes *AA* and *AB* may be due to this synonymous SNP. Although synonymous mutations lead to no change in the amino acid sequence, they may impact on gene function in other ways. There is some evidence that they may have a direct impact on gene function because they affect mRNA stability, folding, and translation; protein folding; and miRNA-based regulation of expression [25]. It is also possible that they have an effect on wool traits due to being linked to functionally important variation outside of the region investigated in this study. Further investigation of proximal genes and other inter-genic regions near *KRTAP6-3* is required to confirm this.

The research in this and previous studies [9,14] has revealed seven allelic variants of ovine *KRTAP6-3*. It is possible that more variants may be found, if more sheep from more breeds are investigated. This suggests that ovine *KRTAP6-3* is highly polymorphic. Furthermore, variation in ovine *KRTAP6-3* has been found to be associated with variation in three fibre diameter-associated wool traits: MFD, FDSD, and PF. These are important characteristics in determining the quality and price of wool. This would suggest that variation in ovine *KRTAP6-3* may be used in genetic selection to improve fibre diameter-associated traits. In the context of this, selection for *G* may lead to the production of finer wool with a narrow distribution of fibre diameter and a reduced ‘coarse edge’, whereas selection for *B* may lead to coarser wool with reduced uniformity.

The possibility exists that the effects observed for *KRTAP6-3* may be due to its linkage to other *KRTAPs* on the same chromosome. Twelve other *KRTAPs* have been identified on sheep chromosome 1 near *KRTAP6-3*, including four other members of the KAP6 family [9], *KRTAP7-1* [22], *KRTAP8-1* [22], *KRTAP8-2* [23], *KRTAP11-1* [13], *KRTAP13-3* [26], *KRTAP15-1* [24], *KRTAP22-1* [8], and *KRTAP24-1* [27]. Regardless of the potential for linkage, the extent of the genetic variation and its associations with MFD, FDSD and PF suggests that ovine *KRTAP6-3* may have potential as a gene-marker for fine wool production.

## Figures and Tables

**Table 1:** The association of *KRTAP6-3* genotype and various wool traits.

Trait ^1^		Mean ± SE ^2^		*p*^3^
	*AA* (*n* = 238)	*AB* (*n* = 17)	*AG* (*n* = 105)	
GFW (kg)	2.14 ± 0.11	2.23 ± 0.15	2.11 ± 0.12	0.631
CFW (kg)	1.62 ± 0.09	1.66 ± 0.12	1.57 ± 0.10	0.454
Yield (%)	75.4 ± 1.63	74.3 ± 2.18	74.0 ± 1.81	0.220
MSL (mm)	85.0 ± 3.23	83.9 ± 4.32	86.9 ± 3.60	0.483
MSS (N/ktex)	21.5 ± 2.13	22.7 ± 2.84	21.0 ± 2.36	0.732
MFD (µm)	19.0 ± 0.46 ^b^	20.8 ± 0.61 ^a^	18.1 ± 0.51 ^c^	**<0.001**
FDSD (µm)	4.04 ± 0.16 ^b^	4.41 ± 0.21 ^a^	3.79 ± 0.18 ^c^	**<0.001**
CVFD (%)	21.2 ± 0.59	21.1 ± 0.79	20.8 ± 0.66	0.552
MFC (°/mm)	89.1 ± 4.20	91.4 ± 5.61	85.3 ± 4.66	0.197
PF (%)	1.89 ± 0.79 ^b^	4.88 ± 1.06 ^a^	0.76 ± 0.88 ^c^	**<0.001**

^1^ GFW: Greasy Fleece Weight; CFW: Clean Fleece Weight; MSL: Mean Staple Length; MSS: Mean Staple Strength; MFD: Mean Fibre Diameter; FDSD: Fibre Diameter Standard Deviation; CVFD: Coefficient of Variation of Fibre Diameter; MFC: Mean Fibre Curvature; PF: Prickle Factor (percentage of fibres over 30 microns); ^2^ Estimated marginal means and their standard errors (SE), and *p*-values derived from general linear models. Means within rows that do not share a superscript letter are different at *p* < 0.05; ^3^ *p-*values < 0.05 are in bold.
